# Opportunities for the Qualified Practitioner

**Published:** 1907-09-07

**Authors:** 


### The Graduate's Work.

### OPPORTUNITIES FOR THE QUALIFIED PRACTITIONER.

The study to which the somewhat clumsy and cumbrous adjective "post-graduate" has been prefixed as specially distinguishing it from that with which the student is concerned before he qualifies, is one which, though it has, perhaps, a definite commencement has its ending only with the life of the student. Who can define what post-graduate study means? It is easy enough to say that it is definable as being "an interest in and a study of medicine and its allied sciences, not for examination purposes, but with the object of broadening and extending essentials already acquired by a graduate in medicine who is desirous of equipping himself more fully for his work in life." Such an explanation is no definition, and, indeed, it is unnecessary to attempt to define what we mean by the term "post-graduate study" when we preface all remarks about it by postulating that the medical practitioner, as the true scientist, must ever be a student, ever ready to say "I do not know but I wish to learn," and ever eager to make the most of such opportunities as are thrown in his way to perfect such knowledge as he may already have attained to, or to open to him new fields as interesting and as profitable to traverse as those through which he marched in his school days.

### The Student Graduate.

Yet there are general practitioners, or men newly qualified, who take a more practical and somewhat less idealistic view of the nature of post-graduation studies. "Knowledge for its own sake" is not a motto which is usually honoured in practice in our utilitarian age. In his pre-graduation days the ordinary medical student has had a surfeit of useless, and for purposes of private practice in after life, wholly unprofitable work in amassing knowledge not even for its own sake, but often merely for the sake of satisfying examiners in a syllabus drawn up with a most illogical disregard for the comparative value of things and subjects. Once qualified the stress and strain of private practice are in many cases too great to permit of much leisure or inclination for special study. The specialism he desires is eminently practical. Hints that may be of value to him in diagnosis and treatment—less pathology and more therapeutics—to know what strychnine and camphor and ergot do for the sick man, not what they may achieve in the laboratory on the frog's heart, the unstriped muscle of the newt's intestine, or the comb of a barn-door fowl. That is the kind of work the qualified man will say appeals to him. To the few who are idealistic enough to see that "utility only" is a catchword which has its limitations and drawbacks, there are other attractive branches of study, directly or correlatively connected with the main subjects by means of which the practitioner wins his daily bread and butter. But it is not with these that we have to deal in writing about the post-graduate's work. An easy, and for all practical purposes, an accurate classification of the types of men who seek work and study after they have passed their final qualifying examination is that which divides them into three categories or groups. Firstly, there are practitioners who, having qualified and obtained a diploma, wish to obtain further qualifications or degrees; secondly, there are those who wish to "specialise" in some subject or in a variety of subjects, in the hope that by so doing they may improve their chances of success. Lastly, but by no means the least important class, are those general practitioners who wish to amplify their experience, "to brush up their work," as they term it, and to become better acquainted with the latest developments in the various branches of the profession.

### Degrees and Accessory Qualifications.

The practioner who wishes to add to his list of qualifications or degrees has a more or less definite programme in front of him, from which the demands of the various examining bodies admit little, if any, divergence. These demands are clearly defined and exactly stated in most instances, and a summary of them will be found on another page in this issue. For the most part the facilities for study afforded by the various special classes, both at medical schools, by private tutors, and at certain hospitals, suffice for this class of practitioner, especially if, as is usually the case, study at such special courses is augmented by diligent home reading and a review of the candidate's own experience as given in his note- and case-books.

### Specialising.

The man who wishes to improve himself in certain subjects, either to become proficient in such subjects or because, as is very often the case, he has had little opportunity of studying more than their mere elements during his student days, stands on a somewhat different footing. We may take it that he rarely intends to specialise so exclusively as to become a consultant in that particular branch to which he devotes himself, for we are dealing throughout these articles with general practitioners, not with students who are ambitious of becoming leading lights of the profession. To the former a certain amount of extra proficiency in some speciality is often of inestimable advantage. A good knowledge of eyes or of ears contributes greatly towards the reputation of a man in a country practice where his colleagues are not averse to calling in his aid on occasions when the specialist, pure and simple, is unavailable. In a certain sense such a course of post-graduate special study is imperative. Few students, it is safe to say, have put on forceps, or done a venesection, or give an anæsthetic on their own responsibility before qualifying, and the majority of men gain their first opportunities in that way when they are doing locum tenencies, or, if fortunate enough to secure one, holding a house appointment. But for more advanced special work something more than casual instruction, however sound and however practical, is necessary. The graduate will have to attend special courses, select special hospitals, and hold special appointments to obtain the requisite degree of confidence in his work and the necessary knowledge and experience which alone can warrant such confidence. Such a man must plan out his own programme, seeking for himself the best means of obtaining exactly what he wants. The facilities which are at hand are so numerous and so varied that no adequate enumeration of them can be given here. The process of selection and elimination is in some cases difficult, especially where the claims of different schools appear equally strong. In most cases the practitioner's own hospital will offer him greater scope in the way of securing appointments than he can elsewhere obtain: in others he may find a few months spent at a special hospital (such as, for instance, the Rotunda, the fever hospitals, or a sanatorium) of distinct benefit. Nearly all special institutions offer opportunities and chances to the man who is really "keen." At most of them minor appointments, entailing a three or six months' period of responsibility and permitting of solid work, are available. Such posts are of great value, and the practitioner who wishes to devote more attention to any special branch is strongly recommended to consider them very carefully.

### The Conscientious Graduate.

The requirements of the third type—that is, "the conscientious practitioner who, alive to his own limitations, wishes to do the best for his reputation, his patients, and his pocket"—are so real and so varied that they demand some detailed consideration, and we can do no better than repeat what we have already stated on a former occasion when dealing with this subject. The practitioner requires, in the first place, a practical experience which he can readily and directly apply to his patients' needs. He may be forgiven a not unnatural aversion to academic lectures, remembering his early student days and the work which was of the greatest service in his examination—the out-patient department. He requires that which books and the lecture theatre do not supply—namely, personal and practical acquaintance with the diseases of everyday life. In academic lectures he sees an expensive luxury which he cannot afford at present. His passive toleration of them is only equalled by his enthusiasm for a practical "tip" or a useful prescription. He enjoys a "good tip," and justifiably, feeling that he is acquiring in a concrete form the experience of a practical teacher. Laboratory and high clinical research he admires from a distance, but he welcomes a knowledge of the quickest, simplest, and most reliable method of demonstrating a "typhoid reaction," a blood-count, a sugar or albumin test, or the staining of a tubercle bacillus. He sees in his own practice occasional cases of aural suppuration, but he wishes to see groups of them in different stages and degrees of severity, to become familiar with their differential diagnosis, complications, and sequelæ, and the perfected details of treatment. He wants to know when and how to syringe the ears, to inflate the tympanic cavity, how to see the glottis, and to be able to appreciate what he does see. These things he views from a business aspect, and realises that contact with a practical teacher is a "solid asset" to him which can be applied in his own practice, yielding a substantial return in cash and "kudos." He wishes to be "up to date," yet while fully appreciating the value and fascination of an expert's lecture, he prefers to absorb it through the medical journals at his leisure; but he cannot see the "cream" of a large out-patient clinic in the weekly journal. "I know," says he, "that in functional aphonia the voice may be at once restored by the interrupted current, but in my last case I did not succeed; therefore I want to learn by practical demonstration why I failed." He has no desire to become an expert laryngologist, oculist, or aurist, but he wishes to become more expert. He prefers the conversational demonstration in which he takes a direct personal share to the more impersonal lecture. Finally, he requires his teaching so arranged as to interfere as little as possible with his regular work, and at a moderate cost pecuniarily.

### "Brushing Up."

In addition, there is one type of man who has already had a fairly wide experience of clinical work, but who is sufficiently enthusiastic to consider the time spent in theoretical and practical study as not being wasted. He is essentially the man who wants to add to and improve his knowledge; to brush up and freshen his acquaintance with laboratory methods, with new instruments and appliances, and with the latest developments which have a practical value for him, and may render him real service in his work. It is only of recent years that this type of professional student has been fully recognised, and already there are so many excellent schools which make a special feature of their courses for him that he will not need to go very far in his search for aids and facilities. The Polyclinic and the West London Schools are excellent for such students, and the practitioner's own school will always be ready to welcome him back, and to admit him once more into the confraternity of students, to make him free once more of lectures, ward demonstrations, and, usually for a small fee, of practical laboratory work as well. In general the eager practitioner who returns to his old haunts in quest of more information is a valuable asset to the medical school, not often, unfortunately, recognised as such. His practical experience is often of service in the wards, and his influence on younger students is invariably for good. On the other hand, by mixing with men so much his juniors in years and in professional understanding, he also gains in no small degree. For that reason it is of mutual benefit to himself and to his school if he elects to return. Schools specially designed for graduates have, of course, their advantages, and every graduate who undertakes special study should decide upon becoming a member of one of these schools. There he meets with men of his own years, with tastes very often similar, and with aspirations often analogous, to his own. There, too, he may revive old friendships and knit new ones. But by belonging exclusively to such a graduate club, as one may term it, and by neglecting the opportunities offered him by his own hospital, we incline to believe that he loses certain advantages which would have been of material value to him.

